# Blinded by and Stuck in Negative Emotions: Is Psychological Inflexibility Across Different Domains Related?

**DOI:** 10.1007/s42761-022-00145-2

**Published:** 2022-10-07

**Authors:** Ella K. Moeck, Jessica Mortlock, Sandersan Onie, Steven B. Most, Peter Koval

**Affiliations:** 1 Melbourne School of Psychological Sciences, The University of Melbourne, Parkville, VIC 3010 Australia; 2 School of Psychology, University of New South Wales, Sydney, Australia; 3 The Black Dog Institute, Randwick, Australia; 4 Emotional Health for All Foundation, Java, Indonesia; 5 Faculty of Psychology and Educational Sciences, KU Leuven, Belgium

**Keywords:** Emotional inertia, Repetitive negative thinking, Emotion-induced blindness, Psychological inflexibility

## Abstract

Psychological inflexibility is theorized to underlie difficulties adjusting mental processes in response to changing circumstances. People show inflexibility across a range of domains, including attention, cognition, and affect. But it remains unclear whether common mechanisms underlie inflexibility in different domains. We investigated this possibility in a pre-registered replication and extension examining associations among attentional, cognitive, and affective inflexibility measures. Participants (*N* = 196) completed lab tasks assessing (a) *emotion-induced blindness*, the tendency for task-irrelevant emotional stimuli to impair attention allocation to non-emotional stimuli; (b) *emotional inertia*, the tendency for feelings to persist across time and contexts; and global self-report measures of (c) *repetitive negative thinking*, the tendency to repeatedly engage in negative self-focused thoughts (i.e., rumination, worry). Based on prior research linking repetitive negative thinking with negative affect inertia, on one hand, and emotion-induced blindness, on the other, we predicted positive correlations among all three measures of inflexibility. However, none of the three measures were related and Bayes factors indicated strong evidence for independence. Supplementary analyses ruled out alternative explanations for our findings, e.g., analytic decisions. Although our findings question the overlap between attentional, cognitive, and affective inflexibility measures, this study has methodological limitations. For instance, our measures varied across more than their inflexibility domain and our sample, relative to previous studies, included a high proportion of Asian participants who may show different patterns of ruminative thinking to non-Asian participants. Future research should address these limitations to confirm that common mechanisms do not underlie attentional, cognitive, and affective inflexibility.

**Supplementary Information:**

The online version contains supplementary material available at 10.1007/s42761-022-00145-2.

Optimal psychological functioning requires constant adaptation to changing situational demands (Kashdan & Rottenberg, 2010). For instance, although it is functional to be fearful and hyperaware of your surroundings when walking alone at night (Keltner & Gross, 1999), it is equally important for fear and hyperawareness to subside after safely arriving home. Emotions that persist when they are no longer situationally relevant may reflect *psychological inflexibility*, a psychopathology hallmark involving difficulty adjusting thoughts, feelings, and behaviors in response to changing circumstances (Kashdan & Rottenberg, 2010; Stange et al., 2017). Researchers have investigated several forms of psychological inflexibility, but it remains unclear whether different forms of inflexibility are driven by common underlying mechanisms. We sought to answer this question by examining associations among attentional, cognitive, and affective inflexibility measures.

Previous studies have linked *repetitive negative thinking*, a form of cognitive inflexibility that encompasses rumination (Nolen-Hoeksema et al., 2008) and worry (Borkovec & Inz, 1990), with *emotional inertia*, a form of affective inflexibility where feelings are resistant to change over time (Kuppens et al., 2010). Repetitive negative thinking and emotional inertia have been hypothesized to be two sides of the same coin, both driven by impaired disengagement from negative stimuli and events (Brose et al., 2015; Koval et al., 2012, 2015). Whereas repetitive negative thinking is typically assessed using global questionnaires, emotional inertia is operationalized as the autoregressive slope of repeatedly sampled affect (Suls et al., 1998). Repetitive negative thinking and negative affect inertia are positively related in European samples, both when inertia is captured in daily life (e.g., Brose et al., 2015) and in the lab (Koval et al., 2016; as in the current study). Taken together, these and other studies suggest cognitive and affective inflexibility may be mutually reinforcing or driven by common processes (Gilbert et al., 2019; Stefanovic et al., 2021).

Research on a phenomenon known as *emotion-induced blindness* suggests that the same processes underlying cognitive and affective inflexibility might also drive attentional inflexibility. In an emotion-induced blindness paradigm, a series of non-emotional (neutral) images are rapidly presented in a centrally located stream. One of these neutral images—the target—is rotated to the left or right. On some trials, an emotional or neutral distractor appears within the image stream. Participants’ ability to detect the orientation of the target image is impaired when it follows an emotional, relative to a neutral, distractor (Most et al., 2005). In other words, the emotional image temporarily “blinds,” hence the term emotion-induced blindness. Emotion-induced blindness is most robust when the neutral target appears shortly after the emotional distractor (e.g., 100-200 ms; Kennedy & Most, 2015), and tends to dissipate as the latency between the emotional and neutral stimuli increases. Thus, researchers propose that emotion-induced blindness reflects early perceptual competition between emotional and non-emotional stimuli (e.g., Most & Wang, 2011).

But prior research suggests emotion-induced blindness may also be influenced by individual differences in cognitive and affective inflexibility. Consistent with this idea, emotion-induced blindness has been linked with individual differences in difficulty terminating (but not initiating) episodes of worry assessed in daily life in a predominantly Caucasian sample (Berenbaum et al., 2018), and with self-reported repetitive negative thinking (using similar measures to the current study; Kennedy & Most, 2015; Onie & Most, 2017, but see Onie & Most, 2021). In an experiment with U.S. undergraduates, Haddara et al. (2019) found that emotion-induced blindness persisted at longer latencies (e.g., 400–700 ms) following an anticipatory anxiety induction (i.e., the threat of electric shock). This finding suggests that heightened anxiety—which characterizes episodes of worry and repetitive negative thinking—may make it more difficult for people to flexibly shift their attention away from emotional and towards neutral stimuli.

No previous research has examined associations between negative affect inertia and emotion-induced blindness. However, given that both constructs have been linked with repetitive negative thinking (Koval et al., 2016; Onie & Most, 2017), we reasoned that negative affect inertia and emotion-induced blindness may also be associated. This association would support the proposal that a common mechanism underlies inflexibility across attentional, cognitive, and affective domains (e.g., Gilbert et al., 2019). Domain-general psychological inflexibility may confer increased risk of psychopathology by leading to a tendency to get stuck in self-perpetuating cycles of negative thoughts, feelings, and behaviors (Robinson et al., 2006; Stange et al., 2017). It is thus important to understand whether such domain-general inflexibility exists.

In the current study, we investigated whether measures of three different forms of psychological inflexibility are related, and therefore may be influenced by common underlying processes. We used the same measures as the studies we sought to replicate: undergraduate participants completed an emotion-induced blindness task (as in Onie & Most, 2017), an emotional film-task to assess negative affect inertia (as in Koval et al., 2016), and global worry and rumination questionnaires to assess repetitive negative thinking (as in Onie & Most, 2017; Koval et al., 2016 assessed rumination only). In line with these studies, we hypothesized that repetitive negative thinking would be positively associated with (a) emotion-induced blindness (H1; Onie & Most, 2017) and (b) negative affect inertia (H2; Koval et al., 2016). Extending these studies, we predicted emotion-induced blindness would be positively associated with negative affect inertia (H3). Consistent with the notion that individual differences in emotion-induced blindness at longer latencies may reflect inflexible attention allocation, for both H1 and H3, we predicted stronger associations with emotion-induced blindness on trials that had a longer interval between the emotional distractor and the neutral target.

## Method

We pre-registered hypotheses and data collection procedures (https://aspredicted.org/kb7mu.pdf). Data and analysis code are publicly available (https://osf.io/e6rwk/).

### Participants

We powered this study based on the correlation effect sizes reported in the studies we sought to replicate. These correlations were *r* = .34 for emotion-induced blindness and repetitive negative thinking (rumination and worry; Onie & Most, 2017), and *r* = .19 for negative affect inertia and rumination (Koval et al., 2016). We therefore designed our study to be sufficiently powered to detect effect sizes around *r* = .25. Using G*Power 3.1 (Faul et al., 2009), we determined that a sample size of *N* = 180 would allow us to detect correlational effects of this magnitude with 80% power and an alpha level of .01.^1^ We therefore aimed to recruit 200 participants to allow for attrition or data loss.

We recruited 209 first-year University of Melbourne psychology students who participated for course credit. We excluded six participants due to technical errors during the film-task. We excluded a further seven participants based on their performance on the emotion-induced blindness task. The emotion-induced blindness task includes trials with and without distractors. In line with our pre-registration and Onie and Most (2017), we excluded seven participants who scored more than 3 SDs below mean accuracy on distractor trials, and more than 3 SDs below mean accuracy collapsed across all trials (i.e., distractor and no-distractor trials). The final sample (*N* = 196) comprised 154 female, 41 male, and 1 non-binary participant, aged 17 to 36 (*M* = 19.13, SD = 2.09). Participants reported their ethnicity as Chinese (49.5%), Caucasian (24%), non-Chinese Asian (22.4%), or “other” (4.2%; including African, Middle Eastern, Hispanic). The University of Melbourne Human Research Ethics Committee approved this study (application 1953918.1). Data were collected in 2019, prior to the COVID-19 pandemic.

### Measures

#### Repetitive Negative Thinking

As in Onie and Most (2017), we assessed repetitive negative thinking using the Penn State Worry Questionnaire (Meyer et al., 1990) and the Ruminative Response Scale (Treynor et al., 2003). The Penn State Worry Questionnaire comprises 16-items (e.g., “my worries overwhelm me”). Participants rated how typical each item is of them from 1 (*not at all typical of me*) to 5 (*very typical of me*). The Ruminative Response Scale comprises 22-items representing three subscales: reflection (e.g., “Write down what you are thinking and analyze it”), brooding (e.g., “Think about a recent situation, wishing it had gone better”), and depression-related (e.g., “Think about how passive and unmotivated I feel”). Participants rated how often they do each item when feeling down, sad, or depressed on a 4-point scale (1 = *almost never*, 2 = *sometimes*, 3 = *often*, 4 = *almost always*). Total scores on the Penn State Worry Questionnaire and Ruminative Response Scale showed excellent internal consistency in our sample (see Table 1). Scores on both scales correlated at *r* = .55 and were combined into a repetitive negative thinking measure (Samtani et al., 2021), also highly reliable in our sample (*α* = .93). To compare our sample’s scores on these measures with Onie and Most’s (2017) sample (Worry: *M* = 47.44, SD = 13.67; Rumination: *M* = 48.03, SD = 11.78), we present sum scores in Table 1, but used mean scores for all analyses.

**Table 1:** Descriptive statistics and reliability estimates of key variables

Variable	*M* (SD)	Actual range	Possible range	Reliability
Repetitive negative thinking (RRS & PSWQ total)	104.87 (20.41)	48–156	48–163	.93^a^
Rumination (RRS total)	51.14 (10.96)	27–83	22–88	.89^a^
Worry (PSWQ total)	53.72 (12.21)	21–80	16–80	.92^a^
EIB lag-2 negative accuracy (%)	68.2 (11.4)	33.3–91.7	0–100	.73^c^
EIB lag-2 neutral accuracy (%)	74.9 (13.2)	21.7–98.3	0–100	.84^c^
EIB lag-4 negative accuracy (%)	76.4 (15.5)	16.7–96.7	0–100	.89^c^
EIB lag-4 neutral accuracy (%)	81.3 (15.9)	16.7–100	0–100	.92^c^
Negative affect inertia (autoregressive slope)	.181 (.208)	-	-	Within: .79 Between: .89^b^

*RRS*, Ruminative Response Scale; *PSWQ*, Penn State Worry Questionnaire; *EIB*, emotion-induced blindness

^a^Cronbach’s alpha; ^b^Alpha values estimated using multilevel structural equation modelling (Geldhof et al., 2014); ^c^Spearman Brown split-half correlation calculated with 5,000 permutations (Parsons, 2020). Sums are provided; dividing the repetitive negative thinking scores by 38, RRS scores by 22, and PSWQ scores by 16 provides mean scores

#### Negative Affect Inertia

We used the same film-task as Koval et al. (2016; Study 2), which is a slightly modified version of the task developed by Koval et al. (2013). The task—programmed in Psychopy (Peirce et al., 2019)—comprised 10 film-clips (four negative, four positive, two neutral; all < 3 min). These film-clips were selected from a validated database (Schaefer et al., 2010) and shown in the following fixed order: negative, negative, neutral, positive, neutral, negative, positive, positive, negative, positive (as in Koval et al., 2013, 2016). Immediately after viewing each film-clip (regardless of film-clip valence), participants rated their momentary^2^ positive (happy, amused) and negative (disgusted, angry, sad, nervous) affect (from 1 = *not at all* to 100 = *very much*). Participants then viewed a 20-s neutral image (a recovery period) before again rating their affect. In addition to rating affect twice after each film-clip (once before, once after the recovery period), participants rated their affect before the first film-clip (baseline), yielding 21 affect ratings total. The baseline rating was made shortly after completing a neutral practice trial, which was not included in analyses. For more film-clip task details, see Koval et al. (2013, 2016).

As pre-registered, we calculated momentary negative affect during the film-task by taking the mean of angry, sad, and nervous ratings at each measurement occasion. The negative affect scale showed good multilevel reliability: *α*_within_ = .79, *α*_between_ = .89 (Geldhof et al., 2014).^3^ We also calculated momentary positive affect (see supplementary material Table S7). The validity of the film-task is supported by previous studies showing that negative affect inertia based on the film-task correlates positively with negative affect inertia in daily life modelled using experience-sampling data (Koval et al., 2013, 2015) and is similarly associated with rumination (cf. Koval et al., 2012, 2016).

#### Emotion-Induced Blindness

We used the same images and rapid serial visual presentation task as Onie and Most (2017), programmed in MATLAB with Psychophysics Toolbox extensions (Brainard, 1997; Pelli, 1997). Each trial comprised a stream of 17 landscape and architectural images presented for 100 ms each. Participants were instructed to indicate (via keypress) the rotation (90° left or right) of a target image embedded within this stream. Also embedded within most trials was a negative (e.g., medical trauma, violence) or neutral (e.g., animals, people) distractor image. Images were originally sourced from the International Affective Picture System (Lang et al., 2005) and other public sources. In a pilot study, Kennedy and Most (2012) had 12 participants rate this image set on valence (1 = *very negative*, 9 = *very positive*) and arousal (1 = *low arousal*, 9 = *high arousal*). Relative to the neutral images, the negative images were significantly lower in valence, *M*_Negative_ = 1.73, *SD*_Negative =_ 0.53; *M*_Neutral_ = 5.01, *SD*_Neutral_ = 0.45), *t*(11) = 35.0, *p* <.001, and higher in arousal, *M*_Negative_ = 6.06, *SD*_Negative =_ 0.68; *M*_Neutral_ = 3.20, *SD*_Neutral_ = 0.55, *t*(11) = 24.4, *p* <.001. The distractor image appeared either 200 ms (lag 2) or 400 ms (lag 4) before the target image. We included lag 2 and 4 trials because emotion-induced blindness may be more sensitive to individual differences at longer latencies (i.e., at lag 4 vs. lag 2), although previous research suggests the effect is reliable at both lags (Ciesielski et al., 2010). There were 300 trials in total: 240 had distractors (120 negative, 120 neutral) and 60 did not (baseline trials). We calculated split-half reliabilities using the *splithalf* package (Parsons, 2020) in *R.* Split-half reliabilities on the emotion-induced blindness task ranged from good to excellent: *r* = .73 to .92 (see Table 1).

We operationalized emotion-induced blindness as the difference in target detection accuracy following negative vs. neutral distractors, separately for lag 2 and lag 4 trials. This operationalization matches several studies examining whether emotion-induced blindness is sensitive to individual differences (Berenbaum et al., 2018; Haddara et al., 2019; Most et al., 2005; Olatunji et al., 2013; Onie & Most, 2017). However, other operationalizations also exist, such as using accuracy following negative distractors only (Onie & Most, 2017) or comparing accuracy following negative distractors at different lags to index emotional disengagement specifically (Kennedy & Most, 2015; Olatunji, 2021). We report analyses using these alternative operationalizations in the supplementary materials (Tables S1-S2).

### Procedure

Participants attended the lab in groups of two to seven but completed the study individually, seated in separate cubicles. After providing consent, participants provided demographic information and completed several global self-report measures, including the repetitive negative thinking questionnaires.^4^ Lastly, participants completed the emotion-induced blindness and inertia tasks in counterbalanced order (determined by coin toss before each group’s session). All tasks and questionnaires were presented on a 21.5-in. monitor with 1920 × 1080 resolution and 60-Hz refresh rate. Consistent with the studies we sought to replicate (Koval et al., 2016; Onie & Most, 2017); participants’ head position was not fixed. Debriefing procedures concluded the 60-min session.

### Statistical Analyses

Consistent with our pre-registration, we used correlations to examine the relationship between repetitive negative thinking and emotion-induced blindness (H1). Because our focus was on estimating the relationship between two variables, this analysis differs slightly from Onie and Most (2017), who sought to test whether emotion-induced blindness predicted repetitive negative thinking over-and-above another form of attentional bias using multiple regression. Also consistent with our pre-registration, we used multilevel models to examine how negative affect inertia relates to repetitive negative thinking (H2) and emotion-induced blindness (H3). This approach matches prior work examining the relationship between inertia and rumination (Koval et al., 2012, 2016). We decided post-hoc to supplement the results of these frequentist analyses with Bayes factors (as in Onie & Most, 2017). We included Bayes factors because it is not possible to determine whether non-significant findings represent the absence of an effect, or that the data cannot distinguish between the null and alternative hypotheses based on *p* values alone (Dienes, 2014; Quintana & Williams, 2018). By directly comparing relative evidence for and against the null hypothesis, Bayes factors provide evidence for three possibilities: that the data (1) favor the alternative hypothesis (Bayes factors over 1), (2) favor the null hypothesis (Bayes factors <1), or (3) favor neither hypothesis (Bayes factors ~ 1; Dienes, 2014). In the current study, we quantified the level of evidence indicated by the Bayes factors (BF_10_) following Wetzels et al. (2011): for the alternative hypothesis (decisive >100, very strong = 30–100, strong = 10–30, substantial = 3–10, anecdotal = 1–3) and for the null hypothesis (anecdotal = 1–0.3, substantial = 0.3–0.1, strong = 0.1–0.03, very strong = 0.03–0.01, decisive < 0.01).

We used JASP statistical software (JASP Team, 2020) to estimate correlations between repetitive negative thinking (and separately worry and rumination) scores and emotion-induced blindness at lag-2 and lag-4, i.e., to test H1. We calculated Bayes factors in JASP with default Cauchy priors (Rouder et al., 2009). We used Mplus 8.6 (Muthén & Muthén, 2017) to test whether negative affect inertia positively related with repetitive negative thinking (H2) and emotion-induced blindness (H3). Specifically, we estimated multilevel autoregressive models with data from the emotional film-task (as in Koval et al., 2016). At level-1, we regressed each participant’s momentary negative affect at time *t* onto their “lagged” negative affect at time *t–*1, with higher autoregressive slopes reflecting greater inertia (Suls et al., 1998). We person-mean centered lagged negative affect, meaning that autoregressive slopes were purely within-persons, and the level-1 intercept represented mean negative affect across the film-task (Hamaker & Grasman, 2015). At Level-2, the intercept and autoregressive slope were allowed to vary randomly across participants and were regressed onto standardized repetitive negative thinking (H2; and separately worry and rumination) or emotion-induced blindness (H3) scores. We calculated Bayes factors for our multilevel parameter estimates using the approach outlined by Wagenmakers (2007): we compared the Bayesian information criterion (BIC) from each model including the hypothesized effect with the criterion obtained from a null-hypothesis model where the hypothesized association was constrained to zero. Our original (pre-registered) power analysis was based on estimating single-level correlations rather than multilevel interaction effects. Thus, we used Murayama et al.’s (2022) multilevel power calculator to verify that our sample size (*N* = 196, cluster size = 21) was sufficient to detect a cross-level interaction equivalent to *r* = .20 with 80% power (*α* =.05).

## Results

### Preliminary Analyses

We first calculated descriptive statistics for each measure (see Table 1). Rumination and worry levels were higher than those reported by Onie and Most (2017), also in an Australian university sample. Rumination levels in both the current study and Onie and Most exceeded those found in a European university sample by Koval et al. (2016; *M* = 39.38). There were no obvious floor or ceiling effects, and variance in rumination and worry levels—indicated by the standard deviations in Table 1—was comparable to the studies we sought to replicate (Koval et al., 2016; Onie & Most, 2017). The average negative affect autoregressive (inertia) slope was positive and significant, indicating that negative affect tended to carry over from moment-to-moment (see Table 1).

To test whether participants showed emotion-induced blindness, we ran a 2 (distractor-type: negative, neutral) × 2 (lag: 2, 4) repeated measures ANOVA, following previous emotion-induced blindness research (e.g., Onie & Most, 2017). Participants were less accurate following negative (*M* = 72.3, SD = 12.6) than neutral (*M* = 78.1, SD = 14.1) distractors; a main effect of distractor type, *F*(1, 195) = 188.75, *p* <.001, ŋ_p_^2^ = .49. Participants performed worse when distractors appeared 200 ms (*M* = 71.5, SD = 11.5) than 400 ms (*M* = 78.9, SD = 15.3) before the target, a main effect of lag, *F*(1, 195) = 164.19, *p* <.001, ŋ_p_^2^ = .46. There was a significant distractor-type × lag interaction, *F*(1, 195) = 5.63, *p* = .019, ŋ_p_^2^ = .03 (see Table 1). Post hoc *t* tests with Bonferroni correction revealed emotion-induced blindness was stronger at lag 2, *t*(195) = −11.34, *d* = 0.81, than lag 4, *t*(195) = −8.29, *d* = 0.59. Thus, participants showed emotion-induced blindness, and this effect was stronger with shorter intervals between the distractor and the target.

### Hypothesis Testing

**Fig. 1:**
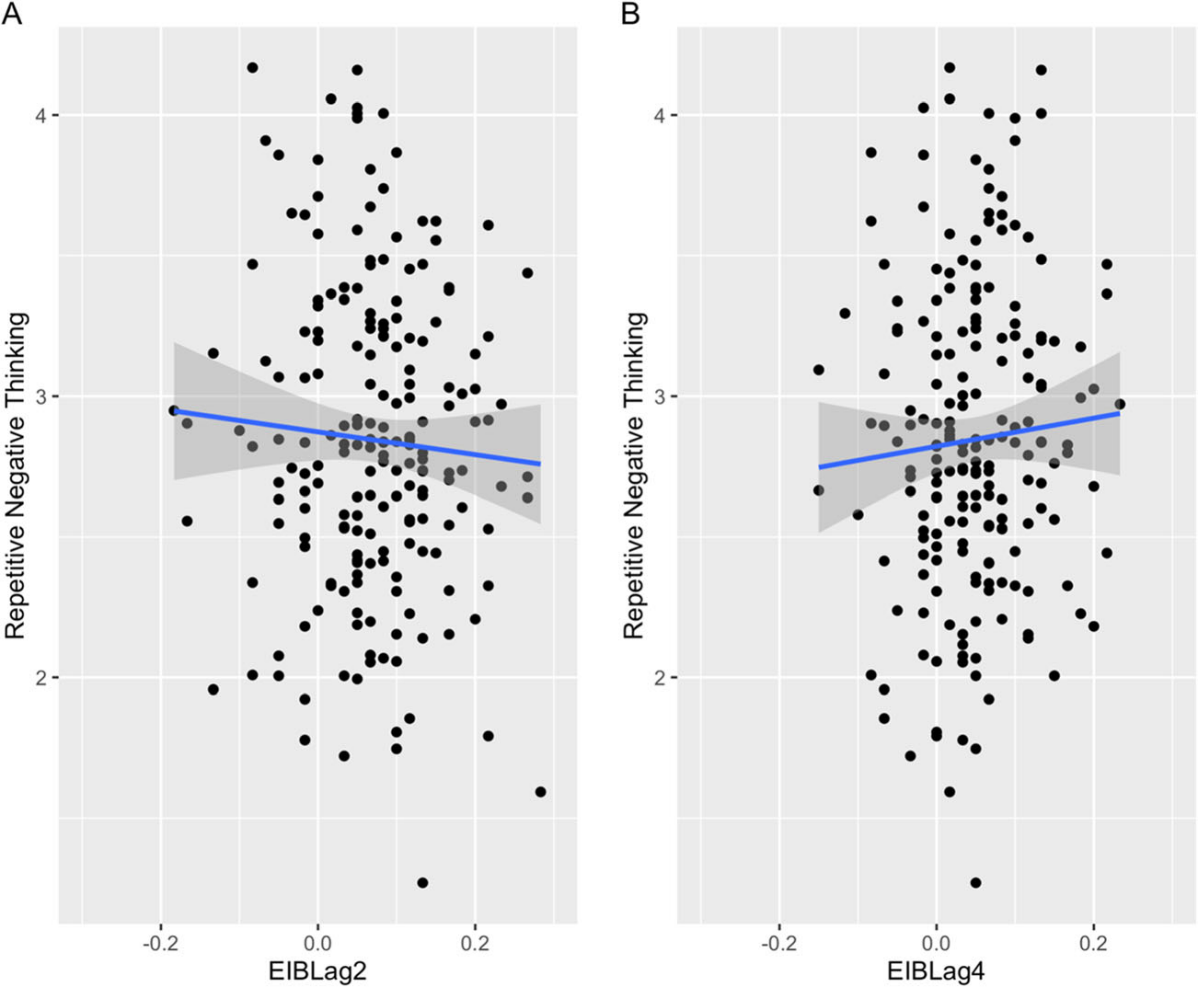
Scatterplots of repetitive negative thinking with emotion-induced blindness (EIB) at lag 2 (panel A) and lag 4 (panel B)

Having established that participants showed the expected effects and variability on all three measures, we turn to hypothesis testing. Contrary to H1, we found no evidence that repetitive negative thinking, rumination, or worry correlated with emotion-induced blindness at lag 2 or lag 4 (Table 2). Figure 1 displays scatterplots of participants levels of repetitive negative thinking and emotion-induced blindness at lag 2 and lag 4. Bayes factors ranged between 0.09 and 0.15 (Table 2), indicating either substantial (values between 0.3 and 0.1) or strong (values between 0.03 and 0.01) support for the null hypothesis (Wetzels et al., 2011). These findings suggest independence between emotion-induced blindness and all aspects of repetitive negative thinking, failing to replicate Onie and Most’s (2017) findings.

**Table 2:** Correlations (*r*, [95% CI]) between emotion-induced blindness at lag 2 and lag 4 and the repetitive negative thinking composite, rumination, and worry

	EIB-lag 2	EIB-lag 4
Repetitive negative thinking (RRS & PSWQ)	−.03, [−.17, .11] BF_10_ = 0.096	.03, [−11, .17] BF_10_ = 0.099
Rumination (RRS total)	−.07, [−.21, .07] BF_10_ = 0.150	.01, [−.13, .15] BF_10_ = 0.090
Worry (PSWQ total)	.01, [−.13, .15] BF_10_ = 0.090	.04, [−.10, .18] BF_10_ = 0.107

No correlations were statistically significant. *RRS* Ruminative Response Scale, *PSWQ* Penn State Worry Questionnaire, *EIB* emotion-induced blindness, *BF* Bayes factor

**Fig. 2:**
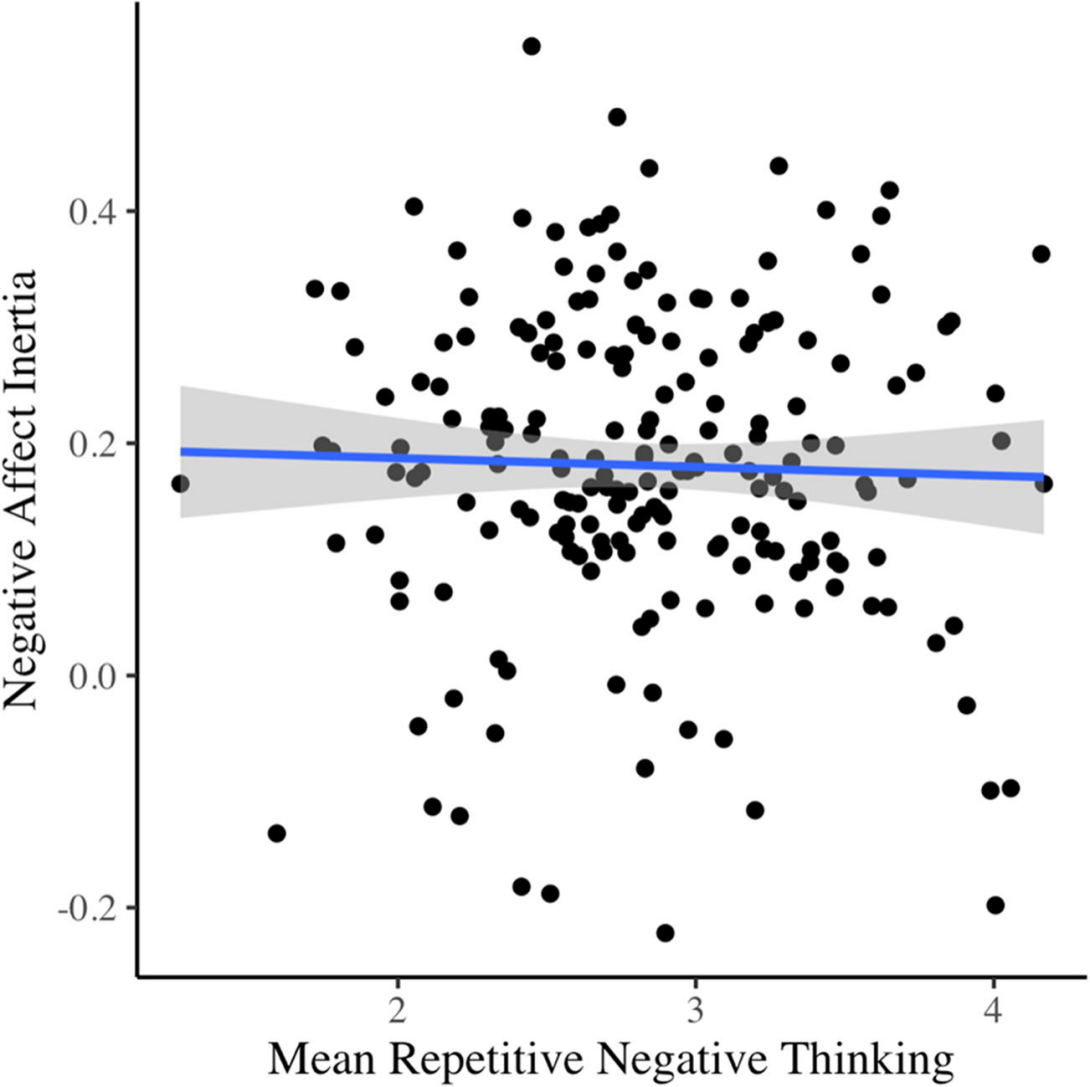
Scatterplot of repetitive negative thinking and negative affect inertia (autoregressive slope)

Contrary to H2, we found no evidence that repetitive negative thinking, rumination, or worry predicted negative affect inertia (Table 3). The Bayes factor for all three models was .017. This Bayes factor indicates very strong support for the null hypothesis (values between 0.03 and 0.01; Wetzels et al., 2011) of no association between negative affect inertia and any aspect of repetitive negative thinking (see Fig. 2). Indeed, repetitive negative thinking did not significantly correlate with the autoregressive inertia slope (*r* = −.04, *p* = .339, 95% [CI −.24, .16]). Therefore, we failed to replicate Koval et al. (2016). Despite not being associated with negative affect inertia, repetitive negative thinking, worry, and rumination were associated with higher mean negative affect levels, shown by the significant associations with the intercept reported in Table 3.

**Table 3:** Results of multilevel autoregressive models estimating associations between raw negative affect inertia, repetitive negative thinking, and emotion-induced blindness

	Association with intercept (mean negative affect)				
			*95% CI*		
*Outcome/predictor*	*Estimate (SE)*	*p value*	*LL*	*UL*	
Repetitive negative thinking	**2.899 (1.033)**	**.005**	**0.874**	**4.924**	
Rumination	**2.400 (1.107)**	**.03**	**0.230**	**4.570**	
Worry	**2.678 (0.986)**	**.007**	**0.746**	**4.611**	
EIB lag-2	−1.232 (0.965)	.202	−3.122	0.659	
EIB lag-4	−0.987 (0.989)	.318	−2.926	0.952	
	Association with inertia slope (cross-level interaction)				Bayes factors
Repetitive negative thinking	−0.009 (0.025)	.717	−0.058	0.040	0.017
Rumination	−0.007 (0.024)	.764	−0.054	0.039	0.017
Worry	−0.009 (0.025)	.722	−0.058	0.040	0.017
EIB lag-2	0.041 (0.022)	.062	−0.002	0.085	0.095
EIB lag-4	0.006 (0.022)	.764	−0.036	0.049	0.017

*N* = 196 for all analyses; estimates in bold are statistically significant at *p* < .05. *EIB*, emotion-induced blindness

**Fig. 3:**
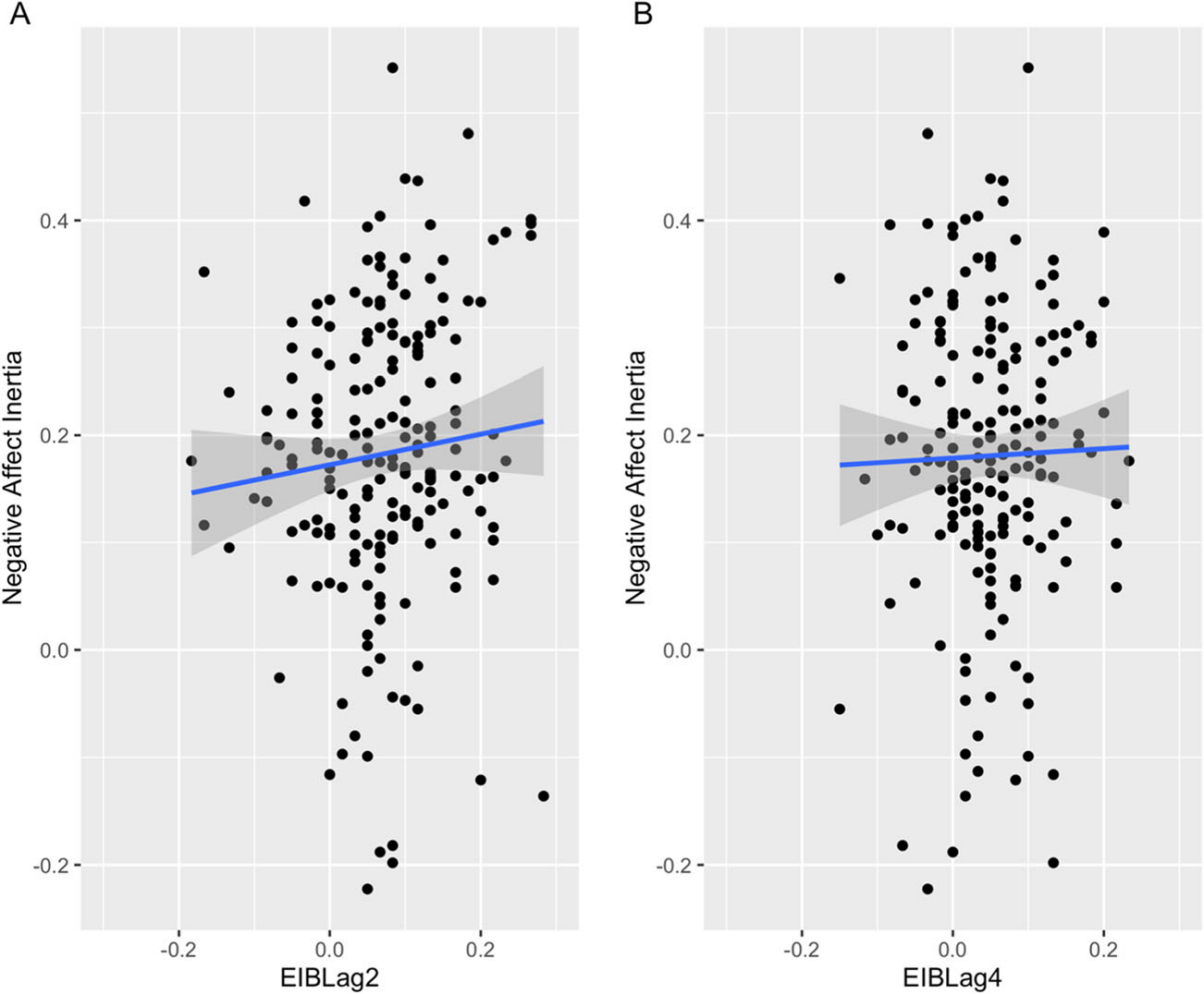
Scatterplots of negative affect inertia (autoregressive slope) and emotion-induced blindness (EIB) at lag 2 (panel A) and lag 4 (panel B)

Finally, contrary to H3, we found no evidence for an association between negative affect inertia and emotion-induced blindness at lag 2 or lag 4 (see Table 3 and Fig. 3). Although the model for negative affect inertia and lag 2 emotion-induced blindness was positive with a *p* value of .06, the Bayes factor of 0.095 indicated strong evidence for the null hypothesis. Indeed, the Bayes factors for this series of models were 0.017 and 0.095, indicating either strong (values between 0.1 and 0.03) or very strong (values between 0.03 and 0.01) support for the null hypothesis. There was also no association between mean negative affect and emotion-induced blindness at lag 2 or lag 4.

### Supplementary Analyses

We ran additional, non-preregistered analyses to ensure methodological and analytic decisions did not explain our null results. The results of these analyses appear in the supplemental materials. First, to confirm that our null findings related to emotion-induced blindness were not due our specific operationalization of emotion-induced blindness, we re-ran the emotion-induced blindness analyses using two alternative indices: accuracy following negative distractors only (as in Onie & Most, 2017) and the difference in accuracy on negative distractor trials between lag 4 and lag 2 (as in Kennedy & Most, 2015). For the correlations between repetitive negative thinking and emotion-induced blindness (Table S1), we found substantial or strong evidence for the null hypothesis. For the multilevel models testing the association between negative affect inertia and emotion-induced blindness (Tables S2), we found very strong evidence for the null hypothesis. Therefore, our results remained consistent regardless of emotion-induced blindness operationalization.

Second, to confirm that our null findings for negative affect inertia were not confounded by mean levels and variability in negative affect, we re-ran multilevel models testing H2 and H3 using within-person standardized negative affect ratings (Table S3), which hold constant individual differences in mean levels and variability of affect (Koval et al., 2013, 2016). Third, because autoregressive models assume stationarity (i.e., that the mean and variance are stable over time; Jongerling et al., 2015), we also re-ran our multilevel models with detrended negative affect scores (Table S4). Fourth, we re-ran models for negative affect inertia (raw and standardized) including disgust (Table S5) to ensure that excluding disgust from momentary negative affect did not change our results. Our findings did not change with these variations in operationalization of negative affect inertia.

Fifth, given that the brooding component of rumination is considered particularly maladaptive (e.g., Hasegawa et al., 2014) and has been found to correlate most strongly with emotional inertia (Koval et al., 2012), we re-ran analyses that involved repetitive negative thinking using only the brooding subscale of the Ruminative Response Scale to represent rumination (Table S6). Our H1 and H2 findings did not change. We also conducted analyses for positive affect inertia and found no associations with either repetitive negative thinking or emotion-induced blindness (Table S7).

Finally, given that a majority (71.9%) of our sample identified as Asian whereas previous research on inertia and rumination has predominantly involved European samples, we ran additional exploratory analyses testing whether ethnicity moderated the relationship between repetitive negative thinking and inertia. The results of these analyses are presented in supplementary material Table S8. We recommend these results be interpreted with caution given the current study was not designed to test for cross-cultural differences.

## Discussion

To investigate whether inflexibility in attention, cognition, and affect may be driven by common underlying mechanisms, we examined associations between emotion-induced blindness, repetitive negative thinking, and negative affect inertia. None of our three hypotheses were supported: our findings failed to replicate prior research linking repetitive negative thinking with (a) greater susceptibility to emotion-induced blindness (Onie & Most, 2017) and (b) higher negative affect inertia (Koval et al., 2016). We also found no support for our novel hypothesis that emotion-induced blindness and negative affect inertia would be positively related. We calculated Bayes factors to quantify the strength of evidence against our hypotheses (Dienes, 2014), which consistently indicated strong evidence for no association among these three measures. Supplemental analyses revealed that these findings were robust to a range of methodological and analytic decisions. At face value, our findings suggest that responses on measures of inflexibility in attention, cognition, and affect are unrelated and are thus unlikely to be driven by common underlying mechanisms. Below we consider several possible interpretations for our findings.

First, we note that the null findings do not appear to be due to floor or ceiling effects nor restricted range. Participants demonstrated similar degrees and variability of emotion-induced blindness and negative affect inertia as in previous studies (Gilbert et al., 2019; Kennedy & Most, 2015; Koval et al., 2016). Our sample reported higher average levels of repetitive negative thinking than those observed in the two studies we sought to replicate (Koval et al., 2016; Onie & Most, 2017), and in other undergraduate samples (e.g., Hasegawa, 2013; Koval et al., 2012; Onie & Most, 2021; Topper et al., 2014). In fact, the current sample’s rumination levels were comparable to participants that were previously or currently depressed (Hasegawa et al., 2014; Koval et al., 2012).

Our finding that emotion-induced blindness did not correlate with repetitive negative thinking fails to replicate Onie and Most (2017), on which we based our first hypothesis. However, the current findings are consistent with a more recent study by Onie and Most (2021), also in an Australian undergraduate sample. In this study, participants (*N* = 99) completed an emotion-induced blindness task and the same measures of rumination and worry as the current study. Participants also completed anxiety and depression scales, and then scores on all questionnaires were combined into a single index encompassing negative affect and repetitive negative thought. Neither scores on this index—nor rumination or worry separately—related to emotion-induced blindness at lags 1, 2, or 8 (Onie & Most, 2021), in line with the current findings.

We consider three possible reasons for these mixed findings across studies, which align with similarly mixed findings regarding the association between emotion-induced blindness and anxiety symptoms (e.g., Berenbaum et al., 2018; Kennedy & Most, 2015; Proud et al., 2020). First, correlations between emotion-induced blindness and psychopathology measures may depend on the personal relevance of the emotional images used in the emotion-induced blindness task. Consistent with this possibility, Olatunji et al. (2013) found combat-exposed veterans with post-traumatic stress disorder showed stronger emotion-induced blindness for combat-related distractors than general negative distractors. Second, correlations between emotion-induced blindness and negative affect measures may be state- rather than trait-dependent. Research showing that inducing state anxiety prolongs the duration of emotion-induced blindness (Haddara et al., 2019) supports this possibility. However, there were no trait anxiety measures included in Haddara et al., making it a future research priority to compare how state and trait levels of a particular domain (e.g., anxiety) relate to emotion-induced blindness within the same sample. Third, relatively small sample sizes may be an underlying reason for the mixed findings. In general, stable correlation estimates—i.e., correlations that approach the true population value with a small confidence interval—require sample sizes approaching 260^5^ (Schönbrodt & Perugini, 2013, 2018). Therefore, the significant correlations found between emotion-induced blindness and constructs like worry, repetitive negative thinking, and anxiety in previous research (e.g., Berenbaum et al., 2018; Kennedy & Most, 2015; Onie & Most, 2017; Proud et al., 2020) may not reflect the true population value. Ongoing research should continue to clarify which emotion-induced blindness parameters (e.g., lag, image content) may be optimally sensitive to which kinds of individual differences, using sufficient sample sizes.

Our finding that negative affect inertia was unrelated to repetitive negative thinking fails to replicate previous studies assessing inertia with the same film-task (Koval et al., 2016) and in daily life (e.g., Brose et al., 2015; Koval et al., 2012). However, participants did show negative affect inertia and we found a positive association between repetitive negative thinking and *mean* levels of negative affect in the film-task. These findings suggest the lack of association with inertia was not due to an absence of meaningful differences in how participants responded to the emotional film-task. Rather, methodological differences might partly explain the inconsistent findings. Here, we focus on two differences: the repetitive negative thinking measures and sample characteristics. Whereas we measured repetitive negative thinking via global worry and rumination questionnaires administered at a single occasion, Brose et al. (2015) used three items (e.g., “Today, I keep thinking about something again and again”) rated daily for 100 days. Therefore, perhaps daily/state measures of repetitive negative thinking relate more strongly to inertia than global assessments of this construct. In line with this possibility, Brose et al. (2015) found that state repetitive negative thinking was more strongly associated with inertia than trait repetitive negative thinking—captured by average daily ratings across the entire study.

However, this methodological difference does not explain why we failed to replicate Koval et al.’s (2016) finding that inertia related to global rumination levels. There are two differences in the characteristics of Koval et al.’s sample and the current sample. First, participants in the current study had higher rumination levels. If there were a curvilinear relationship between inertia and rumination, such that these variables were more strongly positively related at low versus high levels of rumination, our findings (i.e., no association) could be driven by our over-sampling of participants at the higher end of the rumination distribution. Second, and relatedly, our sample mostly self-identified as Chinese or Asian, whereas Koval et al., (2012) and other prior research linking inertia with repetitive negative thinking, has been conducted exclusively with European samples (e.g., Brose et al., 2015; Koval et al., 2012). Relative to Europeans, people from Asian backgrounds show higher levels of rumination (Kwon et al., 2013), yet rumination is less strongly linked with other emotional adjustment measures among Asian participants (Chang et al., 2010). This combination might explain why negative affect inertia was unrelated to repetitive negative thinking in the current study (see supplementary materials for exploratory analyses including ethnicity as a moderator). Future cross-cultural research could directly test this explanation by recruiting equal numbers of Asian and European participants.

More generally, the current findings fit with recent doubts about the value of complex affect dynamic indices in predicting individual differences in personality and psychological adjustment, over-and-above mean levels of affect (Dejonckheere et al., 2019; Wendt et al., 2020). Consistent with this view, we found that the more complex measure of negative affect inertia did not predict repetitive negative thinking, whereas the simpler measure of mean negative affect did.

Finally, there was no association between emotion-induced blindness and negative affect inertia. One possible explanation for this finding is that emotion-induced blindness may reflect an early perceptual impairment (Kennedy & Most, 2012; Most & Wang, 2011; Onie et al., 2020; Zhao & Most, 2019), particularly when there are few items between the emotional distractor and the target image (i.e., at short lags). In line with this idea, when people are “blinded” by an emotional distractor and miss the subsequent target, the target is not perceptually processed (Onie et al., 2020). If emotion-induced blindness is an early, and possibly universal, perceptual impairment, then it is unsurprising that it does not correlate with inflexibility in other domains. Notably, a lack of association between attentional and affective inflexibility measures has also been found using attention tasks that arguably capture distinct processes from those underlying emotion-induced blindness (Gilbert et al., 2019; Iijima et al., 2018). For example, the dot-probe task used by Iijima et al. (2018) may capture spatial attention biases in addition to emotional interference (Onie & Most, 2017), while the attention-shifting task used by Gilbert et al. (2019) includes only neutral stimuli and thus represents valence-neutral attention allocation. Regardless of how attentional inflexibility was operationalized in these studies, it did not correlate with negative affect inertia. In addition, attentional inflexibility uniquely predicts depression onset, separate from other types of inflexibility (Stange et al., 2016). Together, these studies suggest affective and attentional inflexibility are unrelated, perhaps because attentional inflexibility unfolds over a shorter timescale relative to affective (and cognitive) inflexibility.

We acknowledge three key limitations of the current study. First, the study was cross-sectional and therefore tested whether responses on attentional, cognitive, and affective inflexibility measures co-occur. An alternative possibility is that responses on these types of inflexibility measures are related, but the relationship between them emerges over time. Future longitudinal research could examine this possibility and how it might relate to the development of psychopathology like depression symptoms (Stange et al., 2017; Stefanovic et al., 2021). Relatedly, we did not measure participants’ depression or anxiety symptoms in the current study and cannot rule out the influence of such symptoms on our findings. Second, there was inconsistency in whether the measures were process or performance based: the attentional inflexibility measure (emotion-induced blindness) was performance based, while the emotional (negative affect inertia) and cognitive (repetitive negative thinking) inflexibility measures were process based. Combining process- and performance-based measures is a limitation of the broader inflexibility literature (e.g., Gilbert et al., 2019; Stange et al., 2017). Work by Gilbert et al. (2019) suggests future research should address this limitation; they found relationships between responses on process, but not performance-based, inflexibility measures. This limitation could be addressed by choosing components of inflexibility that could feasibly be assessed using either all performance-based, or all process-based, measures. A related limitation is that the scale items used to measure repetitive negative thinking were personally relevant (e.g., “my worries overwhelm me”), while the stimuli used in the emotion-induced blindness and inertia tasks were not. Future research could address this content mismatch by making both tasks more personally relevant. For example, inertia could be measured in daily life in response to personal stressors, while the emotion-induced blindness task could include personally relevant distractors (e.g., Olatunji et al., 2013). Third, our sample included a higher proportion of women (79%) than most prior research on psychological inflexibility (e.g., Brose et al., 2015; Gilbert et al., 2019; Onie & Most, 2017). However, we do not believe this skew fully explains our null results, because Koval et al. (2016; Study 1) found a significant association between rumination and inertia with a similarly skewed sample (86% women). Nevertheless, given women ruminate slightly more than men (e.g., Johnson & Whisman, 2013), future research on psychological inflexibility should aim to use gender-balanced samples.

Despite these limitations, this pre-registered replication and extension study helps to balance the empirical record regarding whether attentional, cognitive, and affective inflexibility are related. Our findings suggest that attentional, cognitive, and affective inflexibility may not stem from the same underlying processes. Rather, distinct processes may underlie inflexibility in these three domains.

## Supplementary Information

ESM 1 (DOCX 54 kb)

## Footnotes

1 Our pre-registration incorrectly states that we would achieve 85% power with *N*=180 and *α* = .01 due to an error in the original write-up of our power analysis.

2 As in Koval et al. (2016), participants were instructed to rate their current emotions honestly and intuitively, rather than how they thought someone should respond to the film.

3 The negative affect scale was also reliable when including disgust (*α*_within_ = .82, *α*_between_ = .93). In the supplementary materials, we report the inertia results including disgust in the negative affect scale (Table S5), as well as results of analyses for positive affect inertia (Table S7).

4 Participants also completed a personality and trait disgust scale, which were not analyzed in the current study.

5 The exact target *N* depends on the expected size of the correlation. Our final *N* of 196 matched the recommended sample size for a correlation of size *r* = .20 (the smallest correlation we powered to detect, based on Koval et al., 2016) to remain within a .15 corridor of stability (r = .050 to .335) with 95% confidence (Schönbrodt & Perugini, 2013).

## Additional Information

### Availability of data and material

The datasets generated during the current study are available at https://osf.io/e6rwk/.

## References

[CR1] Berenbaum H, Chow PI, Flores LE, Schoenleber M, Thompson RJ, Most SB (2018). A test of the initiation–termination model of worry. Journal of Experimental Psychopathology.

[CR2] Borkovec TD, Inz J (1990). The nature of worry in generalized anxiety disorder: A predominance of thought activity. Behaviour Research and Therapy.

[CR3] Brainard DH (1997). The psychophysics toolbox. Spatial Vision.

[CR4] Brose A, Schmiedek F, Koval P, Kuppens P (2015). Emotional inertia contributes to depressive symptoms beyond perseverative thinking. Cognition and Emotion.

[CR5] Chang E, Tsai W, Sanna L (2010). Examining the relations between rumination and adjustment: Do ethnic differences exist between Asian and European Americans?. Asian American Journal of Psychology.

[CR6] Ciesielski BG, Armstrong T, Zald DH, Olatunji BO (2010). Emotion modulation of visual attention: Categorical and temporal characteristics. PLoS ONE.

[CR7] Dejonckheere E, Mestdagh M, Houben M, Rutten I, Sels L, Kuppens P, Tuerlinckx F (2019). Complex affect dynamics add limited information to the prediction of psychological well-being. Nature Human Behaviour.

[CR8] Dienes Z (2014). Using Bayes to get the most out of non-significant results. Frontiers in Psychology.

[CR9] Faul F, Erdfelder E, Buchner A, Lang A-G (2009). Statistical power analyses using G*Power 3.1: Tests for correlation and regression analyses. Behavior Research Methods.

[CR10] Geldhof GJ, Preacher KJ, Zyphur MJ (2014). Reliability estimation in a multilevel confirmatory factor analysis framework. Psychological Methods.

[CR11] Gilbert KE, Tonge NA, Thompson RJ (2019). Associations between depression, anxious arousal and manifestations of psychological inflexibility. Journal of Behavior Therapy and Experimental Psychiatry.

[CR12] Haddara N, Ravid J, Miller EL, O’Hagan M, Caracciolo C, Miskovic V (2019). Anxious anticipation prolongs emotional interference for rapid visual detection. Emotion.

[CR13] Hamaker EL, Grasman RPPP (2015). To center or not to center? Investigating inertia with a multilevel autoregressive model. Frontiers in Psychology.

[CR14] Hasegawa A (2013). Translation and initial validation of the Japanese version of the ruminative responses scale. Psychological Reports.

[CR15] Hasegawa A, Koda M, Hattori Y, Kondo T, Kawaguchi J (2014). Depressive rumination and past depression in Japanese university students: Comparison of brooding and reflection. Psychological Reports.

[CR16] Iijima Y, Takano K, Tanno Y (2018). Attentional bias and its association with anxious mood dynamics. Emotion.

[CR17] JASP Team. (2020). *JASP* (Version 0.13.1) [Computer software].

[CR18] Johnson DP, Whisman MA (2013). Gender differences in rumination: A meta-analysis. Personality and Individual Differences.

[CR19] Jongerling J, Laurenceau J-P, Hamaker EL (2015). A multilevel AR(1) model: Allowing for inter-individual differences in trait-scores, inertia, and innovation variance. Multivariate Behavioral Research.

[CR20] Kashdan TB, Rottenberg J (2010). Psychological flexibility as a fundamental aspect of health. Clinical Psychology Review.

[CR21] Keltner D, Gross JJ (1999). Functional accounts of emotions. Cognition & Emotion.

[CR22] Kennedy BL, Most SB (2012). Perceptual, not memorial, disruption underlies emotion-induced blindness. Emotion.

[CR23] Kennedy BL, Most SB (2015). The rapid perceptual impact of emotional distractors. PLoS ONE.

[CR24] Koval P, Brose A, Pe ML, Houben M, Erbas Y, Champagne D, Kuppens P (2015). Emotional inertia and external events: The roles of exposure, reactivity, and recovery. Emotion.

[CR25] Koval P, Kuppens P, Allen NB, Sheeber L (2012). Getting stuck in depression: The roles of rumination and emotional inertia. Cognition & Emotion.

[CR26] Koval P, Pe ML, Meers K, Koval P, Pe ML, Meers K (2013). Affect dynamics in relation to depressive symptoms: Variable, unstable or inert?. Emotion..

[CR27] Koval P, Sütterlin S, Kuppens P (2016). Emotional inertia is associated with lower well-being when controlling for differences in emotional context. Frontiers in Psychology.

[CR28] Kuppens P, Allen NB, Sheeber LB (2010). Emotional inertia and psychological maladjustment. Psychological Science.

[CR29] Kwon H, Yoon KL, Joormann J, Kwon J-H (2013). Cultural and gender differences in emotion regulation: Relation to depression. Cognition & Emotion.

[CR30] Lang, P. J., Bradley, M. M., & Cuthbert, B. N. (2005). International affective picture system: Technical report A-8. *University of Florida*.

[CR31] Meyer TJ, Miller ML, Metzger RL, Borkovec TD (1990). Development and validation of the Penn State Worry Questionnaire. Behaviour Research and Therapy.

[CR32] Most SB, Chun MM, Widders DM, Zald DH (2005). Attentional rubbernecking: Cognitive control and personality in emotion-induced blindness. Psychonomic Bulletin & Review.

[CR33] Most SB, Wang L (2011). Dissociating spatial attention and awareness in emotion-induced blindness. Psychological Science.

[CR34] Murayama, K., Usami, S., & Sakaki, M. (2022). Summary-statistics-based power analysis: A new and practical method to determine sample size for mixed-effects modeling. *Psychological Methods, Advance Online Publication.*10.1037/met0000330 10.1037/met0000330 35099237

[CR35] Muthén LK, Muthén B (2017). *Mplus user’s guide: Statistical analysis with latent variables, user’s guide*.

[CR36] Nolen-Hoeksema S, Wisco BE, Lyubomirsky S (2008). Rethinking rumination. Perspectives on Psychological Science.

[CR37] Olatunji BO (2021). Emotional induced attentional blink in obsessive-compulsive disorder. Journal of Affective Disorders.

[CR38] Olatunji BO, Armstrong T, McHugo M, Zald DH (2013). Heightened attentional capture by threat in veterans with PTSD. Journal of Abnormal Psychology.

[CR39] Onie, S., MacLeod, C., & Most, S. B. (2020). *Lack of priming suggests early perceptual interference in emotion-induced blindness*. Preprint. 10.1037/emo0001170 36521160

[CR40] Onie S, Most SB (2017). Two roads diverged: Distinct mechanisms of attentional bias differentially predict negative affect and persistent negative thought. Emotion.

[CR41] Onie, S., & Most, S. B. (2021). On the relative sensitivity of spatial and non-spatial measures of attentional bias: Emotion-induced blindness, the dot probe, and gradations in ratings of negative pictures. *Emotion*. 10.1037/emo0000855 34591501

[CR42] Parsons, S. (2020). *Splihalf; robust estimates of split half reliability*. 10.6084/m9.figshare.5559175.v5

[CR43] Peirce J, Gray JR, Simpson S, MacAskill M, Höchenberger R, Sogo H, Kastman E, Lindeløv JK (2019). PsychoPy2: Experiments in behavior made easy. Behavior Research Methods.

[CR44] Pelli DG (1997). The VideoToolbox software for visual psychophysics: Transforming numbers into movies. Spatial Vision.

[CR45] Proud M, Goodhew SC, Edwards M (2020). A vigilance avoidance account of spatial selectivity in dual-stream emotion induced blindness. Psychonomic Bulletin & Review..

[CR46] Quintana DS, Williams DR (2018). Bayesian alternatives for common null-hypothesis significance tests in psychiatry: A non-technical guide using JASP. BMC Psychiatry.

[CR47] Robinson MD, Wilkowski BM, Kirkeby BS, Meier BP (2006). Stuck in a rut: Perseverative response tendencies and the neuroticism-distress relationship. Journal of Experimental Psychology: General.

[CR48] Rouder JN, Speckman PL, Sun D, Morey RD, Iverson G (2009). Bayesian t tests for accepting and rejecting the null hypothesis. Psychonomic Bulletin & Review.

[CR49] Samtani S, Moulds ML, Johnson SL, Ehring T, Hyett MP, Anderson R, McEvoy PM (2021). Higher order repetitive negative thinking is more robustly related to depression, anxiety, and mania than measures of rumination or worry. Cognitive Therapy and Research..

[CR50] Schaefer A, Nils F, Sanchez X, Philippot P (2010). Assessing the effectiveness of a large database of emotion-eliciting films: A new tool for emotion researchers. Cognition & Emotion.

[CR51] Schönbrodt FD, Perugini M (2013). At what sample size do correlations stabilize?. Journal of Research in Personality.

[CR52] Schönbrodt FD, Perugini M (2018). Corrigendum to “At what sample size do correlations stabilize?” [J. Res. Pers. 47 (2013) 609–612]. Journal of Research in Personality.

[CR53] Stange JP, Alloy LB, Fresco DM (2017). Inflexibility as a vulnerability to depression: A systematic qualitative review. Clinical psychology: Science and practice.

[CR54] Stange JP, Connolly SL, Burke TA, Hamilton JL, Hamlat EJ, Abramson LY, Alloy LB (2016). Inflexible cognition predicts first onset of major depressive episodes in adolescence. Depression and Anxiety.

[CR55] Stefanovic, M., Rosenkranz, T., Ehring, T., Watkins, E. R., & Takano, K. (2021). Is a high association between repetitive negative thinking and negative affect predictive of depressive symptoms? A clustering approach for experience-sampling data. *Clinical Psychological Science*, 216770262110094. 10.1177/21677026211009495

[CR56] Suls J, Green P, Hillis S (1998). Emotional reactivity to everyday problems, affective inertia, and neuroticism. Personality and Social Psychology Bulletin.

[CR57] Topper M, Emmelkamp PMG, Watkins E, Ehring T (2014). Development and assessment of brief versions of the Penn State Worry Questionnaire and the Ruminative Response Scale. British Journal of Clinical Psychology.

[CR58] Treynor W, Gonzalez R, Nolen-Hoeksema S (2003). Rumination reconsidered: A psychometric analysis. Cognitive Therapy and Research.

[CR59] Wagenmakers E-J (2007). A practical solution to the pervasive problems of p values. Psychonomic Bulletin & Review.

[CR60] Wendt LP, Wright AGC, Pilkonis PA, Woods WC, Denissen JJA, Kühnel A, Zimmermann J (2020). Indicators of affect dynamics: Structure, reliability, and personality correlates. European Journal of Personality.

[CR61] Wetzels R, Matzke D, Lee MD, Rouder JN, Iverson GJ, Wagenmakers E-J (2011). Statistical evidence in experimental psychology: An empirical comparison using 855 t tests. Perspectives on Psychological Science.

[CR62] Zhao JL, Most SB (2019). Manipulations of distractor frequency do not mitigate emotion-induced blindness. Cognition and Emotion.

