# Supplementary material for: Blinded by and Stuck in Negative Emotions: Is Psychological Inflexibility Across Different Domains Related?
Source: Affect Sci. 2022 Oct 7;3(4):836–48. doi: 10.1007/s42761-022-00145-2 (PMC9540095; doi:10.1007/s42761-022-00145-2)
Supplement: Supplementary file 1 — (DOCX 54 kb) [file 42761_2022_145_MOESM1_ESM.docx]

**Supplementary Materials for “Blinded by and stuck in negative emotions: Is psychological inflexibility across different domains related?”**

Table of Contents

[Emotion induced blindness analyses: Testing alternative emotion-induced blindness operationalizations 2](#_Toc107393108)

[Table S1 2](#_Toc107393109)

[Correlations between repetitive negative thinking and emotion-induced blindness (alternative operationalizations) 2](#_Toc107393110)

[Table S2 3](#_Toc107393111)

[Results of multilevel autoregressive models estimating associations between raw negative affect inertia and emotion-induced blindness (alternative operationalizations) 3](#_Toc107393112)

[Negative affect inertia analyses: Controlling for mean levels and variability in negative affect 4](#_Toc107393113)

[Table S3 4](#_Toc107393114)

[Results of multilevel autoregressive models using standardized negative affect ratings from the film-task to estimate associations between negative affect inertia, repetitive negative thinking, and emotion-induced blindness (EIB) 4](#_Toc107393115)

[Negative affect inertia analyses: Using detrended negative affect scores 5](#_Toc107393116)

[Table S4 5](#_Toc107393117)

[Results of detrended multilevel autoregressive models estimating associations between negative affect inertia, repetitive negative thinking, and emotion-induced blindness (EIB) 5](#_Toc107393118)

[Negative affect inertia analyses: Including disgust in negative affect scale 6](#_Toc107393119)

[Table S5 6](#_Toc107393120)

[Results of multilevel autoregressive models estimating associations between negative affect inertia including disgust, repetitive negative thinking, and emotion-induced blindness (EIB) 6](#_Toc107393121)

[Repetitive negative thinking analyses: Conceptualizing rumination by brooding only 7](#_Toc107393124)

[Table S6 7](#_Toc107393122)

[Results of multilevel autoregressive models estimating associations between raw negative affect inertia and repetitive negative thinking but including only the brooding items from the Ruminative Response Scale 7](#_Toc107393123)

[Positive affect inertia analyses 8](#_Toc107393125)

[Table S7 8](#_Toc107393126)

[Results of multilevel autoregressive models estimating associations between positive affect inertia, repetitive negative thinking, and emotion-induced blindness (EIB) 8](#_Toc107393127)

[Exploratory analyses – Ethnicity as a moderator 9](#_Toc107393128)

[Table S8 10](#_Toc107393129)

[Results of multilevel autoregressive models estimating associations between raw negative affect inertia, repetitive negative thinking, and ethnicity 10](#_Toc107393130)

# Emotion induced blindness analyses: Testing alternative emotion-induced blindness operationalizations

Table S1 displays correlations between repetitive negative thinking measures and alternative operationalizations of emotion-induced blindness: accuracy following negative distractors only (as in Onie & Most, 2017) and the difference in accuracy on negative distractor trials between lag 4 and lag 2 (as in Kennedy & Most, 2015).

## Table S1

### Correlations between repetitive negative thinking and emotion-induced blindness (alternative operationalizations)

|  | Lag-2 Negative | Lag-4 Negative | Lag-4 Negative & Lag-2 Negative Difference |
| --- | --- | --- | --- |
| Repetitive negative thinking (RRS & PSWQ Average) | .008  BF_10_ = 0.090 | -.023  BF_10_ = 0.094 | -.026  BF_10_ = 0.096 |
| Rumination (RRS) | .019  BF_10_ = 0.093 | -.005  BF_10_ = 0.090 | -.049  BF_10_ = 0.113 |
| Worry (PSWQ) | -.011  BF_10_ = 0.090 | -.043  BF_10_ = 0.107 | .016  BF_10_ = 0.092 |

*Note.* No correlations were significant. RRS = Ruminative Response Scale, PSWQ = Penn State Worry Questionnaire.

| Table S2Results of multilevel autoregressive models estimating associations between raw negative affect inertia and emotion-induced blindness (alternative operationalizations) | | | | | | | | | |
| --- | --- | --- | --- | --- | --- | --- | --- | --- | --- |
|  | Association with intercept | | | | Association with NA inertia slope (cross-level interaction) | | | | |
|  |  |  | *95% CI* | |  |  | *95% CI* | |  |
| *Outcome / Predictor* | *Estimate (SE)* | *p-value* | *LL* | *UL* | *Estimate (SE)* | *p-value* | *LL* | *UL* | Bayes Factor |
| Neg-lag 2 | -1.480 (1.042) | .155 | -3.521 | 0.561 | -0.018 (0.022) | .401 | -0.060 | 0.024 | 0.0219 |
| Neg-lag 4 | -1.774 (1.287) | .168 | -4.295 | 0.748 | -0.004 (0.025) | .872 | -0.052 | 0.044 | 0.0162 |
| Neg-lag 4 & Neg-lag 2 Difference | -1.035 (-0.89) | .376 | -3.324 | 1.255 | 0.014 (0.026) | .527 | -0.037 | 0.065 | 0.0192 |

Table S2 displays results of multilevel models for the relationship between inertia and alternative operationalizations of emotion-induced blindness: accuracy following negative (and neutral) distractors as separate predictors and difference in accuracy on negative distractor trials only.

*Note. N* = 196 for all analyses; estimates in bold are statistically significant at *p* < .05

# Negative affect inertia analyses: Controlling for mean levels and variability in negative affect

Table S3 displays multilevel models testing H2 and H3 using within-person standardized negative affect ratings, which hold constant individual differences in mean levels and variability of affect.

## Table S3

### Results of multilevel autoregressive models using standardized negative affect ratings from the film-task to estimate associations between negative affect inertia, repetitive negative thinking, and emotion-induced blindness (EIB)

|  |  | Association with AR(1) slope | | | | |
| --- | --- | --- | --- | --- | --- | --- |
|  |  |  |  | *95% CI* | |  |
| *Outcome / Predictor* |  | *Estimate (SE)* | *p-value* | *LL* | *UL* | Bayes Factor |
| Repetitive negative thinking |  | -0.013 (0.024) | .582 | -0.060 | 0.034 | 0.0198 |
| Rumination |  | -0.017 (0.022) | .456 | -0.060 | 0.027 | 0.0221 |
| Worry |  | -0.009 (0.024) | .716 | -0.055 | 0.038 | 0.0178 |
| EIB lag-2 |  | 0.041 (0.021) | .050 | 0.000 | 0.083 | 0.1098 |
| EIB lag-4 |  | 0.020 (0.020) | .325 | -0.019 | 0.058 | 0.0249 |

*Note. N* = 196 for all analyses; estimates in bold are statistically significant at *p* < .05

# Negative affect inertia analyses: Using detrended negative affect scores

Table S4 displays multilevel models testing H2 and H3 with detrended negative affect scores. We ran these supplementary models because autoregressive models assume stationarity (i.e., that the mean and variance are stable over time).

| Table S4Results of detrended multilevel autoregressive models estimating associations between negative affect inertia, repetitive negative thinking, and emotion-induced blindness (EIB) | | | | | | | | | | |
| --- | --- | --- | --- | --- | --- | --- | --- | --- | --- | --- |
|  | Association with intercept | | | |  | Association with AR(1) slope | | | | |
|  |  |  | *95% CI* | |  |  |  | *95% CI* | |  |
| *Outcome / Predictor* | *Estimate (SE)* | *p-value* | *LL* | *UL* |  | *Estimate (SE)* | *p-value* | *LL* | *UL* | Bayes Factor |
| Raw negative affect inertia |  |  |  |  |  |  |  |  |  |  |
| Repetitive negative thinking | **2.90 (1.04)** | **.005** | **0.86** | **4.94** |  | -0.008 (0.02) | .75 | -0.06 | 0.04 | 0.017 |
| Rumination | **2.40 (1.11)** | **.03** | **0.23** | **4.57** |  | -0.004 (0.02) | .85 | -0.05 | 0.04 | 0.016 |
| Worry | **2.68 (1.00)** | **.007** | **0.73** | **4.63** |  | -0.008 (0.02) | .72 | -0.05 | 0.04 | 0.017 |
| EIB lag-2 negative accuracy | -1.25 (0.96) | .19 | -3.14 | 0.64 |  | 0.033 (0.02) | .12 | -0.009 | 0.07 | 0.056 |
| EIB lag-4 negative accuracy | -0.99 (0.99) | .32 | -2.93 | 0.95 |  | -0.003 (0.02) | .90 | -0.04 | 0.04 | 0.016 |
| Standardized negative affect inertia |  |  |  |  |  |  |  |  |  |  |
| Repetitive negative thinking | — | — | — | — |  | -0.015 (0.02) | .49 | -0.06 | 0.03 | 0.023 |
| Rumination | — | — | — | — |  | -0.015 (0.02) | .46 | -0.06 | 0.03 | 0.022 |
| Worry | — | — | — | — |  | -0.013 (0.02) | .56 | -0.06 | 0.03 | 0.021 |
| EIB lag-2 negative accuracy | — | — | — | — |  | 0.032 (0.02) | .11 | -0.007 | 0.07 | 0.064 |
| EIB lag-4 negative accuracy | — | — | — | — |  | 0.014 (0.02) | .43 | -0.02 | 0.05 | 0.021 |
| *Note. N* = 196 for all analyses; estimates in bold are statistically significant at *p* < .05 | | | | | | | | | | |

# Negative affect inertia analyses: Including disgust in negative affect scale

| Table S5Results of multilevel autoregressive models estimating associations between negative affect inertia including disgust, repetitive negative thinking, and emotion-induced blindness (EIB) | | | | | | | | | | |
| --- | --- | --- | --- | --- | --- | --- | --- | --- | --- | --- |
|  | Association with intercept | | | |  | Association with AR(1) slope | | | | |
|  |  |  | *95% CI* | |  |  |  | *95% CI* | |  |
| *Outcome / Predictor* | *Estimate (SE)* | *p-value* | *LL* | *UL* |  | *Estimate (SE)* | *p-value* | *LL* | *UL* | Bayes Factor |
| Raw negative affect inertia |  |  |  |  |  |  |  |  |  |  |
| Repetitive negative thinking | **2.70 (1.01)** | **.01** | **0.72** | **4.68** |  | -0.01 (0.02) | .65 | -0.05 | 0.03 | .018 |
| Rumination | 2.00 (1.09) | .07 | -0.14 | 4.13 |  | -0.008 (0.02) | .69 | -0.05 | 0.03 | .017 |
| Worry | **2.65 (0.96)** | **.01** | **0.77** | **4.53** |  | -0.01 (0.02) | .67 | -0.05 | 0.04 | .018 |
| EIB lag-2 negative accuracy | -1.20 (0.94) | .20 | -3.03 | 0.64 |  | 0.037 (0.02) | .051 | 0.00 | 0.08 | .095 |
| EIB lag-4 negative accuracy | -0.82 (0.99) | .41 | -2.76 | 1.13 |  | -0.001 (0.02) | .96 | -0.04 | 0.04 | .016 |
| Standardized negative affect inertia |  |  |  |  |  |  |  |  |  |  |
| Repetitive negative thinking | — | — | — | — |  | -0.018 (0.02) | .37 | -0.06 | 0.02 | .026 |
| Rumination | — | — | — | — |  | -0.027 (0.02) | .17 | -0.07 | 0.01 | .046 |
| Worry | — | — | — | — |  | -0.009 (0.02) | .65 | -0.05 | 0.03 | .019 |
| EIB lag-2 negative accuracy | — | — | — | — |  | **0.038 (0.02)** | **.044** | **0.001** | **0.08** | .129 |
| EIB lag-4 negative accuracy | — | — | — | — |  | 0.015 (0.02) | .42 | -0.02 | 0.05 | .022 |
| *Note. N* = 196 for all analyses; estimates in bold are statistically significant at *p* < .05 | | | | | | | | | | |

Table S5 displays multilevel models testing H2 and H3 but with negative affect inertia (raw and standardized) including disgust. We ran these models to ensure that excluding disgust from momentary negative affect did not change our results.

| Table S6Results of multilevel autoregressive models estimating associations between raw negative affect inertia and repetitive negative thinking but including only the brooding items from the Ruminative Response Scale | | | | | | | | | | |
| --- | --- | --- | --- | --- | --- | --- | --- | --- | --- | --- |
|  | Association with intercept | | | |  | Association with AR(1) slope | | | | |
|  |  |  | *95% CI* | |  |  |  | *95% CI* | |  |
| *Outcome / Predictor* | *Estimate (SE)* | *p-value* | *LL* | *UL* |  | *Estimate (SE)* | *p-value* | *LL* | *UL* | Bayes Factor |
| Raw negative affect inertia |  |  |  |  |  |  |  |  |  |  |
| Repetitive negative thinking (brooding for rumination) | **3.247 (1.031)** | **.002** | **1.227** | **5.267** |  | -0.010 (0.025) | .690 | -0.058 | 0.039 | 0.018 |
| Brooding (RRS) | **3.043 (1.122)** | **.007** | **0.844** | **5.242** |  | -0.008 (0.022) | .706 | -0.052 | 0.035 | 0.017 |
| *Note. N* = 196 for all analyses; estimates in bold are statistically significant at *p* < .05 | | | | | | | | | | |

# Repetitive negative thinking analyses: Conceptualizing rumination by brooding only

We re-ran our H1 and H2 analyses but with only the brooding subscale (from the Ruminative Response Scale) included to represent rumination in the Repetitive Negative Thinking composite. For H1, we found no significant correlation between emotion-induced blindness and repetitive negative thinking (lag-2: *r* = -.06, *p* = .403, 95% CI [-.20, .08]; lag-4: *r* = .02, *p* = .772, 95% CI [-.12, .16]), nor brooding alone (lag-2: *r* = -.13, *p* = .075, 95% CI [-.26, .01]; lag-4: *r* = -.01, *p* = .867, 95% CI [-.15, .13]). Table S6 displays multilevel models testing H2. These findings replicate the results from the main H1 and H2 analyses. Therefore, including the reflection items in the rumination measure was not an explanation for the null results.

# Positive affect inertia analyses

Table S7 replicates the multilevel models testing H2 and H3, but with positive affect inertia.

| Table S7Results of multilevel autoregressive models estimating associations between positive affect inertia, repetitive negative thinking, and emotion-induced blindness (EIB) | | | | | | | | | | |
| --- | --- | --- | --- | --- | --- | --- | --- | --- | --- | --- |
|  | Association with intercept | | | |  | Association with AR(1) slope | | | | |
|  |  |  | *95% CI* | |  |  |  | *95% CI* | |  |
| *Outcome / Predictor* | *Estimate (SE)* | *p-value* | *LL* | *UL* |  | *Estimate (SE)* | *p-value* | *LL* | *UL* | Bayes Factor |
| Raw positive affect inertia |  |  |  |  |  |  |  |  |  |  |
| Repetitive negative thinking | -0.37 (1.16) | .75 | -2.64 | 1.90 |  | 0.008 (0.03) | .77 | -0.04 | 0.06 | 0.017 |
| Rumination | -0.58 (1.12) | .60 | -2.78 | 1.62 |  | -0.017 (0.03) | .50 | -0.07 | 0.03 | 0.021 |
| Worry | -0.16 (1.17) | .89 | -2.45 | 2.13 |  | 0.021 (0.02) | .36 | -0.02 | 0.07 | 0.026 |
| EIB lag-2 negative accuracy | 0.57 (0.94) | .55 | -1.28 | 2.41 |  | 0.023 (0.02) | .24 | -0.02 | 0.06 | 0.028 |
| EIB lag-4 negative accuracy | -0.71 (1.05) | .50 | -2.77 | 1.34 |  | -0.004 (0.02) | .86 | -0.05 | 0.04 | 0.016 |
| Standardized positive affect inertia |  |  |  |  |  |  |  |  |  |  |
| Repetitive negative thinking | — | — | — | — |  | -0.007 (0.02) | .76 | -0.05 | 0.04 | 0.017 |
| Rumination | — | — | — | — |  | -0.024 (0.02) | .26 | -0.07 | 0.02 | 0.033 |
| Worry | — | — | — | — |  | 0.006 (0.02) | .76 | -0.03 | 0.05 | 0.017 |
| EIB lag-2 negative accuracy | — | — | — | — |  | 0.026 (0.02) | .18 | -0.01 | 0.06 | 0.037 |
| EIB lag-4 negative accuracy | — | — | — | — |  | 0.008 (0.02) | .68 | -0.03 | 0.05 | 0.018 |
| *Note. N* = 196 for all analyses; estimates in bold are statistically significant at *p* < .05 | | | | | | | | | | |

# Exploratory analyses – Ethnicity as a moderator

We investigated whether ethnicity moderated the association between repetitive negative thinking and inertia. First, given that previous research has suggested that levels and correlates of rumination may differ in Asian vs. non-Asian cultures, and given the ethnic make-up of our sample, we dichotomously coded ethnicity as Asian (*n* = 142) and non-Asian (*n* = 54). We then ran independent samples t-tests to test whether our data supported the claim that people from Asian backgrounds show higher levels of rumination (Kwon et al., 2013). This claim was partially supported: Asian participants showed slightly higher levels of brooding (*M* = 2.41, *SD* = 0.62) than non-Asian participants (*M* = 2.21, *SD* = 0.63), *t*(194) = -2.04, *p* = .043, *d* = 0.33. However, there was no difference between groups (*p* values .30-.99) on worry, overall rumination scores (including brooding and reflection items), or repetitive negative thinking (either with rumination conceptualised using the full Ruminative Response Scale or just the brooding items).

Next, we re-ran the multilevel models testing for the association between repetitive negative thinking and inertia to investigate whether ethnicity (Asian, Non-Asian) moderated this association. In these models, lagged negative affect was a Level 1 predictor, and repetitive negative thinking, ethnicity, and the repetitive negative thinking*ethnicity interaction were all Level 2 predictors. The outcome variable was negative affect. The full results of these models appear in Table S8.

As in our main analyses, repetitive negative thinking was not related to inertia, and this effect did not vary by ethnicity. Given the *t*-tests indicated a significant difference in brooding scores by ethnicity, we re-ran this model including brooding instead of the overall repetitive negative thinking index. Again, we found no evidence that the relationship between inertia and brooding was moderated by ethnicity. Given that the study was not designed to look at ethnicity as a moderator of relationships between inflexibility measures, these analyses may be underpowered (with *n* = 142 participants in one group, and *n* = 54 in the other) and should be interpreted with caution.

| Table S8Results of multilevel autoregressive models estimating associations between raw negative affect inertia, repetitive negative thinking, and ethnicity | | | | | | | | | |
| --- | --- | --- | --- | --- | --- | --- | --- | --- | --- |
|  | Association with intercept | | | |  | Association with AR(1) slope | | | |
|  |  |  | *95% CI* | |  |  |  | *95% CI* | |
| *Outcome / Predictors* | *Estimate (SE)* | *p-value* | *LL* | *UL* |  | *Estimate (SE)* | *p-value* | *LL* | *UL* |
| Raw negative affect inertia | |  |  |  |  |  |  |  |  |
| **Repetitive negative thinking** | **5.74 (2.50)** | **.02** | **0.83** | **10.64** |  | 0.05 (0.05) | .30 | -0.15 | 0.05 |
| Ethnicity | 3.71 (10.15) | .72 | -16.19 | 23.61 |  | -0.29 (0.33) | .39 | -0.93 | 0.35 |
| Repetitive negative thinking * ethnicity | -1.57 (3.51) | .66 | -8.45 | 5.32 |  | 0.11 (0.11) | .30 | -0.10 | 0.33 |
| Raw negative affect inertia | |  |  |  |  |  |  |  |  |
| **Brooding** | **6.21 (2.14)** | **.004** | **2.00** | **10.41** |  | -0.04 (0.04) | .36 | -0.12 | 0.04 |
| Ethnicity | 10.83 (8.97) | .23 | -6.74 | 28.41 |  | -0.22 (0.22) | .33 | -0.65 | 0.22 |
| Brooding * ethnicity | -4.66 (3.60) | .20 | -11.73 | 2.40 |  | 0.11 (0.10) | .24 | -0.08 | 0.30 |
| *Note. N* = 196 for all analyses; estimates in bold are statistically significant at *p* < .05 | | | | | | | | | |
